# A scoping review of Indigenous suicide prevention in circumpolar regions

**DOI:** 10.3402/ijch.v74.27509

**Published:** 2015-03-04

**Authors:** Jennifer Redvers, Peter Bjerregaard, Heidi Eriksen, Sahar Fanian, Gwen Healey, Vanessa Hiratsuka, Michael Jong, Christina Viskum Lytken Larsen, Janice Linton, Nathaniel Pollock, Anne Silviken, Petter Stoor, Susan Chatwood

**Affiliations:** 1 Institute for Circumpolar Health Research, Yellowknife, NT, Canada; 2 Centre for Health Research in Greenland, National Institute of Public Health, Copenhagen, Denmark; 3 Municipality of Utsjoki Health Centre, Utsjoki, Finland; 4 Dalla Lana School of Public Health, University of Toronto, Toronto, ON, Canada; 5 Qaujigiartiit Health Research Centre, Iqaluit, NU, Canada; 6 Southcentral Foundation, Anchorage, AK, USA; 7 Labrador-Grenfell Regional Health Authority, Goose Bay, NL, Canada; 8 John McLean Health Sciences Library, University of Manitoba, Winnipeg, MB, Canada; 9 Sami National Centre for Mental Health, Karasjok, Norway; 10 Centre for Sami Health Research, University of Tromsø – The Arctic University of Norway, Tromsø, Norway

**Keywords:** suicide, circumpolar, Arctic, Indigenous, prevention, interventions

## Abstract

**Background:**

Suicide is a serious public health challenge in circumpolar regions, especially among Indigenous youth. Indigenous communities, government agencies and health care providers are making concerted efforts to reduce the burden of suicide and strengthen protective factors for individuals, families and communities. The persistence of suicide has made it clear that more needs to be done.

**Objective:**

Our aim was to undertake a scoping review of the peer-reviewed literature on suicide prevention and interventions in Indigenous communities across the circumpolar north. Our objective was to determine the extent and types of interventions that have been reported during past decade. We want to use this knowledge to support community initiative and inform intervention development and evaluation.

**Design:**

We conducted a scoping review of online databases to identify studies published between 2004 and 2014. We included articles that described interventions in differentiated circumpolar Indigenous populations and provided evaluation data. We retained grey literature publications for comparative reference.

**Results:**

Our search identified 95 articles that focused on suicide in distinct circumpolar Indigenous populations; 19 articles discussed specific suicide-related interventions and 7 of these described program evaluation methods and results in detail. The majority of publications on specific interventions were found in North American countries. The majority of prevention or intervention documentation was found in supporting grey literature sources.

**Conclusion:**

Despite widespread concern about suicide in the circumpolar world and active community efforts to promote resilience and mental well-being, we found few recorded programs or initiatives documented in the peer-reviewed literature, and even fewer focusing specifically on youth intervention. The interventions described in the studies we found had diverse program designs and content, and used varied evaluation methods and outcomes. The studies we included consistently reported that it was important to use community-based and culturally guided interventions and evaluations. This article summarizes the current climate of Indigenous circumpolar suicide research in the context of intervention and highlights how intervention-based outcomes have largely remained outside of peer-reviewed sources in this region of the world.

Suicide in circumpolar regions is a pressing health policy concern which needs to be understood within the complex socio-economic historical context of Arctic Indigenous peoples. Indigenous communities, government agencies and health care providers have been working towards suicide prevention for decades across the north. Various conferences, task forces and strategies have made it clear that Indigenous communities are demanding solution-focused research on this complex and challenging issue. Such research should recognize Indigenous ownership and inclusion in research and how their stories are being told (1–3) .

Desires for strength-based framing of the issue have led to research that looks at promoting well-being and resiliency, especially among Indigenous youth, who more than any other age group continue to die by suicide in many communities throughout the Arctic (4). As the overview article of this Special Issue by Young and colleagues illustrates, the youth suicide phenomenon should not be generalized across all communities in the Arctic, as substantial variation exists across regions with different historical experiences. These include their extent in retaining Indigenous languages and cultures, and rates of advancement towards self-governance, etc. A circumpolar comparative framework is needed to design, conduct and interpret interventions which would enable lessons to be learned and shared.

The focus on resiliency has to date generated a detailed concept review (5) and a literature review on youth resiliency (6). Kirmayer and colleagues have provided a clear framing of mental health and well-being within the context of Aboriginal and community resiliency (7). Allen and his Alaskan colleagues have extensively investigated and mapped resilience pathways among 5 Indigenous youth populations in Alaska, Canada, Norway and Russia (8).

Framing suicide around protective factors can promote resilience in the face of systemic and political mechanisms that have worked to keep Indigenous populations in adverse conditions across the Arctic. These include residential schools, segregation, isolation, forced relocation, and mainstream health and governance systems that stem from a non-Indigenous worldview. The concept of resilience situates Indigenous suicide within a larger socio-ecological and historical context (9). It is recognized that suicide, mental illness, substance abuse and other health-related challenges are in a large part symptomatic of the long history of colonialism, from which Indigenous communities have only recently begun to emerge from.

Governments, Indigenous communities and health professionals across the north continue to actively discuss how best to design and support suicide prevention programs at the community level. Sharing what has been done and what has worked in different settings is important in informing the direction of future initiatives in the face of sustained suicide prevalence. Unfortunately there seem to be many gaps when attempting to draw a more comprehensive picture of suicide prevention efforts in the northern regions.

In recent years, a small but growing literature on Indigenous youth suicide applicable in the Arctic has emerged, including several major review papers. One systematic review (4) has focused on the protective factors and causal mechanisms that enhance mental health among circumpolar youth, while another by Lehti et al. (10) included mental health, substance use and suicidal behaviour measures in youth across the Arctic. A systematic review of Indigenous youth suicide more globally (11) focused on quantitative outcome measures of suicidal behaviour. These reviews did not include a substantial prevention intervention focus, or found little in the way of intervention-focused articles in their searches. Another relevant systematic review focused specifically on assessing the methodological quality of evaluations of Indigenous suicide prevention programs with a geographic scope that included Australia, United States, Canada and New Zealand (12); however, this review did not cover circumpolar regions outside of North America. Thus questions remain regarding the scope and effectiveness of programs to prevent Indigenous youth suicide in circumpolar regions.

It is clear that there is a need for a current review focusing specifically on suicide intervention initiatives relevant to the circumpolar north. It is our aim to address this gap, in order to inform the design, implementation and evaluation of future Indigenous youth suicide prevention initiatives.

## Methods

We focus our review on interventions, defined broadly as suicide prevention, early intervention or postvention programs, services or policies differentiating Indigenous people (12). Our review is a scoping, rather than a systematic, review. According to Arksey and O'Malley (13), a scoping review aims to map the extent, range and nature of relevant literature in a particular field. A scoping review does not assess the quality of included studies but can be used to assess whether a more comprehensive systematic review is warranted or even feasible. A scoping review can help to identify gaps in the evidence base and summarize a more broad range of research findings (13).

### Focus population

Our population of focus is collectively referred to as Indigenous peoples living in circumpolar regions. This includes diverse cultures and peoples with differing belief systems and languages, who nevertheless share some underlying common values. They inhabit a range of geographical and climatic territories, in villages, towns and cities. Our population is united in particular circumstances of socio-cultural history, where communities have experienced a colonial reality of “significant social and economic transitions and transformations over the last 50 years, stemming from rapid changes in lifestyles and livelihoods across the Arctic” (4). These transitions have been exacerbated by climate change and have contributed significantly to current inequalities in healthcare, education, housing and employment as well as related mental health outcomes (4).

### Research aim

The aim of this scoping review is to document the extent and range of published Indigenous youth suicide prevention efforts situated in the circumpolar north. This effort includes describing the nature of suicide research in the Arctic, noting all programs documented and evaluated in the peer-reviewed literature. A specific focus is taken on suicide prevention, for a 10-year period from 2004 to 2014. The research results are situated within the health policy landscapes of the countries and peoples of the north.

### Search strategy and data extraction

Preliminary academic database searches were conducted with the words: Indigenous, suicide, Circumpolar (or derivatives), prevention, intervention, early intervention, postvention program, service, policy, mental health.

Searching was refined and words included in the search phrase are found in Table I.

**Table I:** Example search equation used in PubMed and Medline based on MeSH, test searches

(intervention* OR “early intervention*” OR postvention OR prevention OR program OR service OR polic*)
AND
(Native* OR Indigenous OR Aboriginal* OR Inuit Or Sami OR Saami OR First Nation OR Metis OR Inuk OR Yup'ik OR Inuviat* OR Yupik* OR Aleut* OR Inupia* OR Alaska Native OR Dene OR Gwichin OR Athabas* OR “American Indian”)
AND
(circumpolar OR polar OR “arctic Canada” OR Nunavut OR Nunavik OR Nunatsiavut OR Inuvialuit OR Yukon OR “Northwest Territories” OR Norway OR Greenland OR Alaska OR Russia OR Sweden OR Finland OR Iceland OR Arctic OR North*)
AND
(suicid*)

Searches were conducted in various electronic databases: Medline, SCOPUS, PubMed, JSTOR, Cochrane Library, Science Citation Index, PsycINFO and Google Scholar. Document databases searched included the High North Research Documents (http://highnorth.uit.no/), the Arctic Health Information Portal (http://www.arctichealth.org/library.php), ministry indicator documents at the Circumpolar Health Observatory (http://circhob.circumpolarhealth.org/online-repository/).

The publication records of key authors and reference lists of selected papers were searched for additional related resources. Google searches and other relevant government websites were searched for supporting grey literature. We also contacted international experts in circumpolar and Indigenous health research, and asked them to identify any studies or reports that were not captured in our electronic searches.

Inclusion criteria were applied in 2 steps to eventually extract evaluation-specific data to inform future prevention interventions. The inclusion criteria were as follows:

### Inclusion criteria

- English-language source (or translated abstract)
- Peer-reviewed journal articles presenting primary research
- Published from 2004 up until March 2014
- Focus on suicide, and includes a distinction of an Indigenous population including Inuit, Yup'ik, Inupiat, Sami, First Nation, or Alaskan Natives located in circumpolar regions or countries only. The eligible circumpolar countries included northern Canada (Yukon, NWT, Nunavut, Nunavik and Labrador), Greenland, Norway, Russia, Finland, Iceland,^1^ Sweden, or Alaska.
- Includes any article mentioning or describing details of a specific intervention, which is *defined as a ‘suicide prevention, early intervention or postvention program, service or policy differentiating Indigenous people’* (12).

### Exclusion criteria

- Non-English-language source (without translated abstract)
- Grey literature
- Anything published before 2004
- Population included in study is not Indigenous from Circumpolar regions or it did not distinguish between Indigenous and non-Indigenous populations
- Does not mention or describe a specific suicide intervention/s

Articles meeting the first 4 inclusion exclusion criteria (N=60) were critically reviewed in their entirety for mentions, descriptions, or evaluations of specific suicide intervention initiatives. Using the publications collected above, a final 2-step process was included in order to collect more detailed information on specific interventions:

- Articles were separated out if they did not have intervention as one of the main focuses of the article or mentioned a specific initiative in passing.
- The remaining articles were then grouped together. These articles were ones that included a detailed evaluation component of a particular intervention (Table II).

**Table II:** Categories used to assess final articles

Data extraction categories	Questions and specifics
Article information	First author, year, publication type
	Study site: community, state/territory/province, country
	Circumpolar Indigenous group differentiated
Study population	Number of participants, timeframes
	Ages, gender, youth vs. adult focus, other
Type of intervention	Program, policy, or service
	Descriptive summary
	Was suicide intervention the focus of the article?
Methodology	Study design, measures used, data collection
	Was the program/data evaluated? How?
	What was the outcome?
	Limitations: what did the authors mention that was not included or missed?
Recommendations	What were the main take home messages?

All related circumpolar literature found during searches including epidemiology, risk and protective factor discussions, and mental health and substance abuse publications that also clearly address suicide were retained as supporting material in order to map the territory of suicide research in the circumpolar region in the context of the recorded interventions. Additionally, available grey literature addressing suicide interventions was also searched in detail solely for comparative reference, including conference proceedings, news bulletins, speeches, workshop reports, research reports, strategies, policies and unpublished articles or program descriptions following the same inclusion criteria.

### Quality review

A scoping review aims to provide a description of the research topic in order to disseminate research findings, summarize and identify potential research gaps (12). No review or analysis of intervention methodology was conducted on the final articles, rather a summary was provided. An independent search librarian conducted an external validation of the literature search, and results were presented to a team of circumpolar researchers and community members twice throughout the process to validate whether the study represented the current research climate or if anything was missed.

## Results

**Fig. 1:**
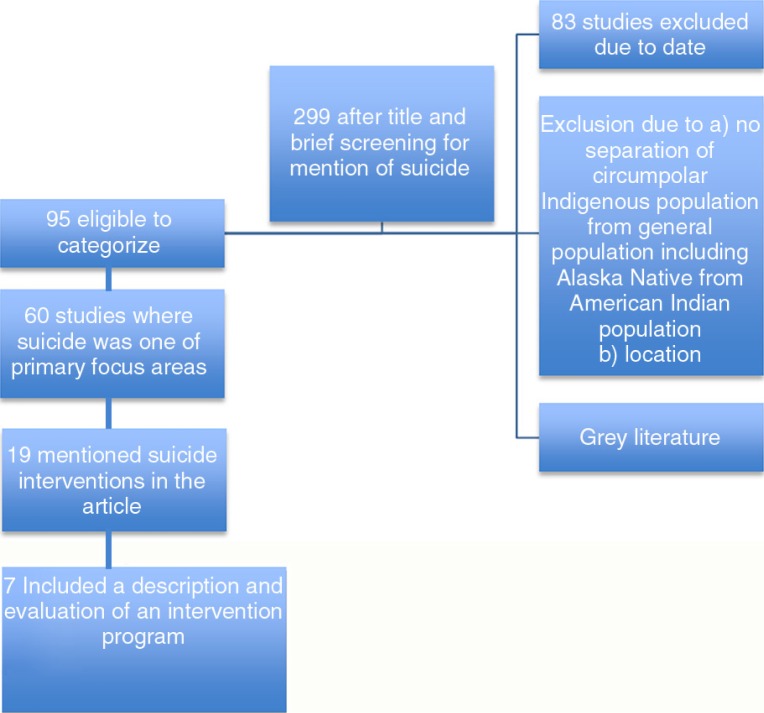
Database search summary. Circumpolar Indigenous suicide 2004–2014.

As shown in Fig. 1, 95 studies were found differentiating Indigenous populations in the circumpolar north with a mention of suicide. Of these, 60 had suicide as a primary focus of the article. Nineteen of these articles included explicit mentions of specific suicide interventions, even if they were just in passing; 8 articles provided detailed evaluation methods and results of specific interventions. However, out of these 8 publications, 1 was excluded from our review (11) as the publication was a systematic review article. Instead we included the 2 original articles featured in this review article, which also met our inclusion criteria (14,15), and refer to the review article as supporting data, resulting in 7 articles which met the final evaluation criteria (see Table III). The other intervention articles were retained for supporting information and related studies were recorded to map the greater extent of the suicide literature in this region of the world.

**Table III:** Published suicide interventions within differentiated Indigenous populations in the Circumpolar North 2004–2014, N=7^a^

First author	Intervention	Description	Location	Indigenous population	Methods	Youth focus
Allen et al. (15)	Program: community prevention	*Qungasvik* toolbox *Elluam Tungiinun* (towards wellness) prevention program	Rural south-western Alaska, US	Yup'ik	Quasi-experimental, interviews, surveys	X
Berman (36)	Program: community prevention	Community alcohol prohibitions – ineffective	178 small Alaskan communities, US	Alaska Native	Poisson regression equations based on statistical data	
DeCou et al. (37)	Other initiatives	Interviews are examined as a suicide intervention	Alaskan University, US	Alaska Native	In-depth semi-structured interview, background questionnaire	
Haggarty et al. (14)	Program: training	CD-ROM training for counsellors and health providers	Nunavut, Canada	Inuit	Multiple choice and opinion questionnaires	
Henry et al. (38)	Program: community prevention	*Qungasvik* toolbox prevention program adapted in 3 communities	Yukon-Kuskokwim region Southwest Alaska, US	Yup'ik	Attendance, video rating, self-report and statistical comparisons	X
Tan et al. (39)	Programs: community prevention	Nunavut *Kamatsiaqtut* help- line	Nunavut, Canada	Inuit	Content analysis, data coding	
Wexler et al. (40)	Programs: community prevention	Digital storytelling suicide intervention initiative by project life	Northwest Alaska, US	Alaska Native	Exit survey, open-ended questions, follow up interviews	X

a Clifford et al. (12) excluded due to being a systematic review.

Specific interventions were classified as either: policies, strategies and services; community prevention programs; or education and training initiatives (11). Most of the circumpolar suicide interventions we found mentioned in the peer-reviewed literature were not described in any detail, but merely mentioned in passing, or listed as brief examples.

**Fig. 2:**
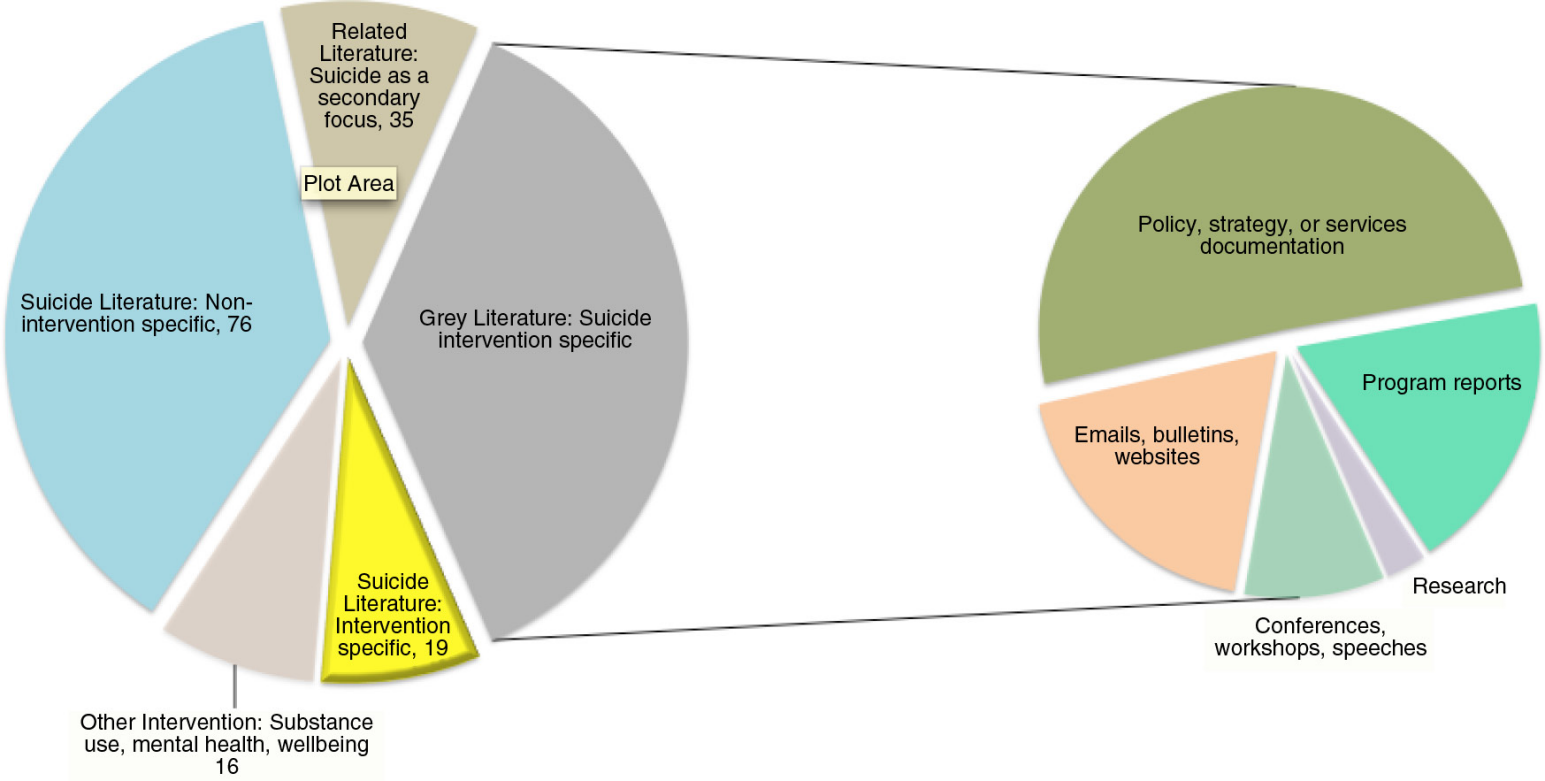
Circumpolar Indigenous suicide literature overview.

A supporting collection of grey literature sources from the review process revealed that the majority of the contextual findings related to specific suicide prevention interventions in the circumpolar north were located in grey literature documents and publications. Out of the 19 peer-reviewed publications that mentioned specific suicide prevention intervention initiatives, 25 distinct interventions were either mentioned in passing or described in detail. This is in comparison to a tally of more than 70 interventions, which were found recorded in grey literature sources across the circumpolar region. It is clear that suicide intervention efforts and recommendations based on actual program outcomes in the circumpolar region have largely remained outside of peer-reviewed publications to date (Fig. 2).

We noted an obvious shift in the suicide literature in this region over the past 20 years. Articles published prior to 2004 tended to employ a deficit-focused approached to the issue, or were highly focused on epidemiological reporting. Definite strides have been made in the recent literature, and there is resounding agreement that culturally-grounded solutions and community-based programs are keys to understanding and approaching suicide prevention. This is consistent with recent political progress in the Arctic around Indigenous rights and governance, including Indigenous populations claiming more control over the research process and how they are portrayed in research and media.

Most of the peer-reviewed literature post-2004 was non-intervention focused (N=76) and comprised of: discussions on suicide, including local and cultural framing (16,17); protective factors (18,19); and recommendations for prevention stemming from participatory or community-based social research (20,21); as well as epidemiological literature looking at statistics and risk factors (22–24) . Of the intervention-focused research (N=19), 6 articles addressed specific policy and service-wide initiatives in the United States, northern Canada, and Greenland (25–30) . Others provided useful discussions on intervention initiatives and evaluation within the circumpolar context (31–35) . As mentioned previously, most articles reviewed merely mentioned various suicide initiatives without providing detailed information on their outcomes.

No intervention specific evaluation data was found published in the peer-reviewed literature from circumpolar countries outside of the United States and Canada (Sweden, Norway, Finland, Greenland, Iceland and the Russian Federation) (see Table III).

Other interventions related to suicide prevention, including substance use, resiliency, or wellness programs were also noted during the searches. Specifically, 16 articles describing mental health, resiliency, or substance use interventions were also found. These articles did not address suicide as a topic and so were not included in the final review.

### Articles which included evaluation data

All studies which included program evaluations, except one (36), showed success in their interventions according to various methods. The evaluation methods varied considerably between interventions, with some using community-level variables, others using surveys and feedback forms, and one completing a detailed inventory and analysis of calls into a call centre. Most of the 7 evaluative studies were situated in Alaska (N=5), with 2 from Nunavut. All clearly addressed the benefits and challenges of evaluative research in a circumpolar context. Additionally we note that 3 of the evaluated interventions had a clear focus on youth (as opposed to young adults or adults).

The one intervention which did not show a measure of success was alcohol restriction in various Alaskan communities (36). Berman (36) found that Alaskan communities which had alcohol prohibitions in place actually had higher suicide rates than communities that did not; however, the overall relational effect was not determined to be statistically significant after controlling for other community characteristics, such as remoteness.

The interventions found in the literature were minimal, highly varied in scope, and exhibited quite different forms of evaluation. A couple notable studies employed unique evaluation methods conducive to community-based cultural suicide prevention programs in a circumpolar context and have insights to offer regarding community-level evaluation and outcomes (15,38).

Clifford et al. and others (11,12) have concluded, based on rigorous systematic reviews, that there is insufficient evidence from published evaluations in Australia, New Zealand, the United States and Canada as to which intervention strategies are most effective for suicide prevention in Indigenous populations. This is largely due to what they state as a lack of strong methodological quality in the analysis and findings of the studies which they have reviewed in these regions (11,12). Since this is a scoping review, we discuss the recommendations based on the intervention-focused articles we did find, regardless of methodological rigour.

### Highlights of promising practices described in the literature

#### Measurement

Evaluation of success within suicide prevention is a challenging and complex endeavour in circumpolar communities (35). Allen and his Alaskan colleagues (15) demonstrate the feasibility of methodological approaches that use community-level variables to measure outcomes in circumpolar suicide prevention research. They mention the utility of measuring community change and mobilization around suicide prevention, to explain the outcome of prevention activities that address suicide in communities (15). Henry et al. (38) also expand upon this same approach within the *Qungasvik* cultural “toolbox” intervention. They outline an innovative framework for the study of function as the unit of analysis in suicide intervention programs (17). They recommend using a dosage of individual protective factor exposure as a common metric across multiple communities, with a focus on assessing the fidelity of the program in sticking with the intended protective factors it was targeting. Such an approach would help evaluate suicide intervention initiatives in locally diverse communities across the circumpolar north, instead of assuming consistency across communities.

Clifford et al. (12) stress that overall, programs should be more rigorously evaluated, including examining impact, and economic costs of interventions. They continue to state that “evaluating Indigenous health interventions is complex and challenging” (p. 7). This raises questions about the evaluation context in remote northern communities, including sample sizes within and across communities, but also the holistic and preventative nature of suicide intervention in Indigenous communities, the capacity for research and the appropriateness of mainstream evaluation standards in evaluating culturally based programs (15,35).

#### Adapt to needs of community

It is highlighted in the literature that prevention and intervention efforts cannot be uniform across Indigenous communities, but must be developed with culturally sensitive and tailored needs (15,38). Allen et al. (15) demonstrate the practice of developing a community-based intervention process that is not a prescriptive manual, but which lays out a process based on community values and common protective factors, which can be adapted. Wortzman (32) echoes this experience when mentioning community-based suicide prevention programs in Alaska, which she describes as involving local Indigenous planning groups who determine the needs and desires of individual communities, and oversee culturally based program activities.

#### Integrating technologies

Wexler et al. (40) show through evaluation that digital storytelling provides a way of promoting personal mastery, highlighting reasons given by youth for living, and strengthening connections with important people in their lives. Haggarty et al. (14) mention the potential for CD-ROM technologies to provide a cost-effective alternative to in-person suicide training in rural and remote regions, regardless of computer know-how. However, they also mention the small sample of their results, and the need to examine video technology in comparison (14).

#### Talking helps

DeCou et al. (37) conclude that discussing the issue of suicide can be an important aspect of suicide prevention for Alaska Native university students, who are survivors of suicide in their communities. They mention that their findings could also offer some assistance to help mitigate concerns raised by research ethics boards over asking questions about suicide during interview research, as they found that actually speaking about suicide had positive benefits for the person being interviewed (33). Students also noted their appreciation of anonymity and being interviewed by someone outside of close-knit daily family and community networks; in fact, they preferred this in order to convey sensitive information (33). Tan et al. (39) investigate the caller details of a crisis line in Nunavut and determined that it was serving its purpose of providing community-based social and emotional support for callers who utilized the service. They found that the crisis line was primarily used by adult females who were in levels of distress around relationship problems, loneliness and boredom (39). They also noted that some users experienced a language barrier on accessing adequate support, for example when an Inuk volunteer was not on shift (39).

#### Screening

Niven (30) encourages routine administration, screening and referral for depression and suicide risk in Alaska village clinics as a means of suicide prevention. However, final outcome-based evaluation data is not provided on the depression and suicide ideation screening form which was collaboratively developed and implemented widely in Alaskan community clinics (30).

#### Consider gender

Tan et al. (39) recommend developing a crisis line specifically dedicated to youth in Nunavut, similar to the children's crisis line in Greenland. They found that overall the crisis line was underused by youth, males and young Inuit males most in need of crisis intervention (39). Berman (36) found that community alcohol restrictions across 178 Alaskan communities were ineffective in preventing suicide among Alaskan Native men.

#### Partnerships with decision makers

Sohota et al. (35) recommend a coordinated effort between community leaders, policy makers and health care providers on developing an evidence base for local culturally based suicide interventions. Dorgan (28) posits that Native American and Alaska Native communities require increased access to mental health services and a variety of innovative suicide prevention initiatives for youth, which should become a priority for all stakeholders in the face of sustained youth suicide rates. This all requires multi-sectoral collaboration between regional and federal governments, Indigenous communities and mental health professionals.

#### Community – researcher partnerships

Clifford et al. (12) recommend strong collaborative partnerships between researchers and Indigenous communities to enable reciprocal exchange of knowledge. Kral et al. (31) highlight that suicide prevention research conducted in First Nations and Inuit communities by external researchers or experts does not always result in direct community benefits to the community, in terms of prevention.

#### Community control

Kral et al. (31) conclude that community control over suicide prevention itself can be effective towards preventing suicide in the Arctic regions. Other researchers confirmed that a community-based participatory intervention process results in greater community ownership and more locally-relevant interventions and outcomes (15,38).

#### Traditional and western knowledge

EchoHawk (27) recommends better integrating Western and Traditional knowledge bases for more culturally appropriate intervention for youth suicide. Allen et al. (15) showed the effective translation of culturally based values into a measurable framing of protective factors analysis, using a quantitative methodology. For example, they show how a subsistence activity of berry picking can promote *ellangneq*, which can be translated into the protective factors of “communally mastery, clear expectations, and praise” (15).

#### Outcome research

Most papers reviewed consistently recommended a need for further studies on outcomes related to suicide prevention intervention. Kral et al. (31) recommend continuous documentation and evaluation of the development process and prevention outcomes in order to inform future initiatives. Bjerregaard and Lynge (26) describe the Greenland Home Rule Government's 2004 plan for suicide prevention, stating that it proposed a number of concrete actions to be taken specifically around the systematic evaluation of the proposed prevention activities. Sahota et al. (35) describe how the United States Substance Abuse and Mental Health Services Administration (SAMHSA)-funded Native Aspirations program in Alaska offers a suite of choices to Alaska Native communities to choose from in regards to implementing existing interventions. They include 3 options for an evidence base: evidence-based practice, practice-based evidence and/or local cultural and spiritual practices, and in so doing, recognize intervention solutions outside of mainstream evidence-based approaches. “Practice-based evidence” includes Alaska Native programs that have been run and have shown successes at the community level, but do not have formal evaluation data recorded (35). They mention that building an evidence base for local and cultural interventions can be a challenging task in the current climate of outcome research (35).

## Discussion

There is a clear lack of concrete information in the peer-reviewed literature on what has actually been done to date for suicide prevention intervention in the circumpolar regions, especially for youth. Even less available are recommendations based on the delivery and outcomes of specific initiatives. Despite the small number of articles found in this review, we identified key articles with information on suicide prevention interventions within a circumpolar context, from Arctic regions across Canada, the United States and Greenland. These have provided valuable insights into the climate of intervention and evaluation in these regions.

Through the process of this review, we have observed the limitations of the peer-reviewed literature itself in realistically capturing suicide intervention in the circumpolar region. This was verified through a supporting grey literature search, which revealed that many community-based interventions had simply not been captured in the primary literature. This is not surprising due to the community-level nature of programs, the challenges involved in mainstream evaluation, and the nature of evaluating success in suicide intervention, an issue embedded in the particular historical, cultural, linguistic and socio-political contexts found in circumpolar regions. Based on their related systemic review, Clifford et al. (12) conclude that the methodological quality of the studies that are published on Indigenous suicide prevention intervention in Canada, the United States, New Zealand and Australia, were “varied with none having consistently strong methodology,” (p. 9) and that there is simply not enough peer-reviewed information available to determine what works for suicide intervention in Indigenous populations. We would take this discussion further within the context of the articles we reviewed, and challenge the current evaluation dialogue within mainstream research climates.

Authors such as Sahota et al. (35) would have us question what rigorous evaluation should look like in an Indigenous circumpolar context, and recommend moving towards more “practice-based evidence,” in which real life practice on the ground in Indigenous communities is also used as a basis for building evidence. This is of course a challenging task, but Sahota et al. (35) recommend 3 concrete steps in this direction applicable to the circumpolar context: funding more evaluations of community-based suicide prevention programs, providing more opportunities for outside evaluation services and broadening the scope of acceptable evidence (35). We find these recommendations highly applicable within circumpolar communities, which are characteristically remote, isolated, and geographically and ideologically removed from the mainstream forms of intervention evaluation. The articles reviewed raise an important debate around the expectations for evaluation in community-level and cultural programming, which are instrumental in harnessing funding for suicide prevention among Indigenous populations. Further to this is the challenge of developing state- or national-level strategies for policy and intervention, and then documenting the interaction between these policy arenas, academic researchers and community action on the ground within Indigenous communities. The nature of and success of suicide intervention is a complex one to reconcile.

### Limitations

There were distinct limitations found within the peer-reviewed literature when trying to capture details on what has been done for suicide prevention in circumpolar regions. No evaluative intervention data was found differentiating Indigenous populations in Sweden, Norway, Finland, Russia and Greenland, though there was information available on suicide rates and trends in these regions. Suicide studies were excluded when they failed to differentiate Indigenous from non-Indigenous populations, and various studies on suicide were eliminated from these regions on this basis alone, which may have contributed to the North American focus of our results, along with a search strategy that was in English. However, a significant effort was made within professional and academic networks within these areas to locate studies that had been missed due to language or bias of North American databases, but no evaluative intervention studies were found.

Another limitation of this review would be the separation of suicide from interrelated topics such as substance abuse, mental health, health promotion, resiliency and wellness research, and physical health. Suicide prevention research in the circumpolar north is highly embedded within a holistic cultural and social–historical climate, and thus limiting the search to suicide itself may have limited findings from related evaluation data. However, care is taken to note these related initiatives, some of which were found during the review, and to acknowledge the importance of this interrelation. The decision to limit publications based on the search term “suicide,” could provide the basis for future discussion on what exactly constitutes suicide prevention intervention, from a more holistic definition and understanding of circumpolar suicide. The benefit of scoping reviews is that they can inform the scope of the current research climate in a specific area. Findings from this study show that the climate of suicide intervention research in the Arctic is sufficiently small enough that further research could expand upon the search criteria, in order to include recommendations based on insights from other related interventions as well.

## Conclusion

It is recommended that more culturally appropriate evaluation and community capacity building around evaluation be supported. Also, further dialogue on what constitutes adequate or useful evidence in suicide intervention, within the context of small northern communities, and within Indigenous worldviews and understandings, should also be stimulated.

We would also encourage researchers interested in suicide prevention to take a more action-focused approach to research. This would entail the inclusion of more detailed information on tangible initiatives and efforts on the ground within the peer-reviewed literature, in order to better inform other communities and regions. Overall, more community-based research focusing on what can be done based on what has (or has not) worked is needed in order to better support our Indigenous communities in the north through prevention intervention. Greater efforts at sharing various successes from across the circumpolar regions could also go a long way towards promoting successful community initiatives, and offer more in the way of resiliency promoting research in the face of suicide-related challenges.

## Footnotes

1 Although Iceland does not have a defined Indigenous population, it was included in the original search to determine the scope of prevention programs in circumpolar regions. In the end, as expected, no articles that met the search criteria were found. Of note, no articles on suicide prevention programs were found in that country.
